# Attitudes toward COVID-19 Vaccine Uptake: A Qualitative Study of Mostly Immigrant Racial/Ethnic Minority Older Adults

**DOI:** 10.3390/geriatrics8010017

**Published:** 2023-01-20

**Authors:** Carla Valero-Martínez, Christopher Martínez-Rivera, Jenny Zhen-Duan, Marie Fukuda, Margarita Alegría

**Affiliations:** 1 Disparities Research Unit, Department of Medicine, Massachusetts General Hospital, Boston, MA 02114, USA; 2 Department of Psychology, Faculty of Social Sciences, Río Piedras Campus, University of Puerto Rico, San Juan, PR 00925, USA; 3 School of Medicine, Ponce Health Sciences University, Ponce, PR 00716, USA; 4 Department of Psychiatry, Harvard Medical School, Boston, MA 02215, USA; 5 Department of Medicine, Harvard Medical School, Boston, MA 02115, USA

**Keywords:** COVID-19, older adults, racial/ethnic minorities, vaccine hesitancy, systemic barriers

## Abstract

(1) Background: Few qualitative studies address diverse older adults’ perceptions of COVID-19 vaccination in the United States, including non-English speakers and immigrant populations. This study aims to understand the attitudes of diverse, primarily immigrant older adults in the U.S. toward the COVID-19 vaccine and its influences on their vaccination decision-making. (2) Methods: The research team conducted semi-structured interviews (*N* = 100) in 2021 focused on understanding ethnically/racially diverse older adults’ perceptions of the COVID-19 vaccine. Interviews were recorded, coded, and analyzed using a thematic analysis approach. (3) Results: Thematic analyses identified three themes. (1) Older adults showed mixed attitudes toward the COVID-19 vaccine associated with information consumed and trust in healthcare systems; (2) health concerns and underlying medical conditions were the most influential factors of vaccine uptake; and (3) systemic barriers and trusted figures impacted vaccination decision-making of older adults. (4) Conclusions: Accessible information in diverse languages tailored to the community’s fears is needed to combat vaccine mistrust. Vaccine rollout programs need to tackle the fear of vaccine side effects. Attitudes of religious leaders, family members, and physicians considerably influenced vaccine uptake, suggesting their role as trusted members for vaccine messaging for older, primarily immigrant adults. Systemic barriers, namely lack of transportation and inaccessible vaccination sites, contributed to vaccine deterrence.

## 1. Introduction

The disproportionate burden of COVID-19 on minority populations [1], predominantly ethnic and racial minorities [2,3] and immigrant populations [4], has highlighted inequities in the U.S. healthcare and social systems. Immigrants are among the minority populations who are particularly vulnerable to the negative impacts of COVID-19. Language barriers and legal challenges can limit many immigrants’ access to timely and quality healthcare [5,6]. Additionally, immigrants are more likely to be essential workers in jobs that put them at greater risk of exposure to COVID-19 [5]. Immigrants are also disproportionately burdened by the negative social and economic impacts of COVID-19 [6] and are excluded from government COVID-19 relief funds and programs [7]. These factors make immigrant populations particularly vulnerable to COVID-19′s harmful effects; this, along with older adults’ increased susceptibility to the virus [8], make vaccination efforts reaching older immigrant populations especially imperative.

Despite nationwide vaccination efforts, there are noticeable differences in vaccination rates among non-Latinx White individuals compared to Black and Latinx populations across most of the United States [9]. Vaccine hesitancy is common among racial/ethnic minorities and immigrants for many reasons [10,11,12]. Historically, racial/ethnic minority adults have lower vaccination rates than non-Latinx White adults [13]. Vaccine hesitancy has also been increasing, even before the COVID-19 pandemic [12]. Hildreth and Alcendor [14] suggest that this skepticism emerges from historical events and systemic racism that may disrupt vaccination uptake. As misinformation increases, the belief that vaccines are ineffective may fuel existing uncertainty among these susceptible populations [15], further increasing their vulnerability to current and future infectious diseases.

Older adults have shown high uptake of the influenza vaccine [16]. Conversely, older immigrants (≥60 years) have lower vaccination uptake for influenza and other vaccines, according to a systematic review [17]. Vaccine hesitancy has been reported among many diverse groups in countries such as India [18], Switzerland [19], Australia, Canada, England, New Zealand, and the United States [20]. Some studies include migrant populations [21], and several studies include older populations [19,22,23,24,25].

Numerous studies have found that there are enablers and barriers to vaccine uptake [20,26], and information on the probability of vaccine effectiveness [27] is an important factor for uptake. Trust in the healthcare system in the host country [20] is another enabler, while a barrier is misinformation [15,20,28,29] or lack of information in immigrants’ primary languages. Work by Fadda and colleagues [19] exploring older adults’ attitudes toward the COVID-19 vaccination in southern Switzerland found that most participants favored vaccination, with concerns being related to safety and the newness of the vaccine. In seeking to understand the perceived benefits and barriers to COVID-19 vaccination of adults with disabilities, illnesses, or comorbidities in Australia, Kaufman and colleagues [26] identified protection from severe disease and the ability to travel as benefits to COVID-19 vaccination and increased uptake. Perceptions of the COVID-19 threat also play a role in uptake [30]. Hesitancy to vaccine uptake also seems fueled by confusion [15] and negative stories that generate distress and uncertainty. Research has also shown that systemic factors contribute to vaccination disparities, including lack of transportation, access to mass vaccination efforts, and inflexible work schedules [9]. Because of the significant risks of non-vaccination, it is essential to understand how diverse populations decide whether to get the vaccine.

Older immigrants’ receptivity to the COVID-19 vaccine is less well-known from qualitative studies with large, diverse older immigrant populations living in different parts of the U.S. and its territories. Several qualitative studies include varied older populations [18,19]. Some are not of older populations but comprise racial and ethnic minorities [30] and a few include undocumented migrants, asylum seekers, and refugees [31]. These studies [18,32,33] emphasize the importance of perceiving COVID-19 vaccine information not as political but as objective, having precise data and knowledge about side effects and the vaccine. There is less qualitative information on non-English speakers and immigrant populations that are racial/ethnic minority older adults and how they feel about getting the COVID-19 vaccine [34,35]. This study contributes to research understanding their attitudes toward vaccine uptake and the influential factors involved in their decision-making process. This information on vaccination is crucial due to diverse older adults’ high risk for COVID-19 morbidity and mortality. Additionally, given that this study included a large sample of racial/ethnic minorities, any evidence of cultural patterns in their responses was explored. Results from this study will help researchers better understand older minoritized adults’ attitudes toward vaccination, which can inform the design of strategies to increase vaccination tailored to their specific concerns and attitudes.

## 2. Materials and Methods

### 2.1. Participants

The research team contacted individuals who had previously participated in the Positive Minds-Strong Bodies clinical trial (PMSB) [36] to obtain their consent for this mixed methods study. The team asked participants to participate in a follow-up interview to understand the decision-making of and factors influencing the uptake of the COVID-19 vaccine in older, mostly immigrant and racial/ethnic and linguistic minority adults. Participants were offered a $40 gift card as compensation if they opted to participate. Participants resided in four states/territories, including Massachusetts, Florida, New York, and Puerto Rico, and were eligible to participate if they could participate in interviews in English, Mandarin, Cantonese, or Spanish. More details of the original study and intervention are offered in Alegría et al. [36].

In the original trial, 307 participants were enrolled and randomized to either the intervention (*n* = 153) or control condition (*n =* 154). Of these 307 participants, 44 were ineligible to complete the COVID-19 follow-up assessment for this study because of the following reasons. (1) Five enrolled participants died before the end of the study and two died during the COVID-19 follow-up data collection; (2) another twenty participants were identified as deceased from the National Death Index as of 31 December 2021; and (3) seventeen participants were unable to complete the COVID-19 follow-up assessment due to medical conditions (e.g., cognitively impaired or severely ill). Of the remaining 263 eligible participants, 165 (62.7%) were reached and completed the COVID-19 follow-up assessment.

Because qualitative interviews could only be conducted among participants who completed the COVID-19 follow-up assessment, we consider the 165 who completed the COVID-19 follow-up assessment as those who were eligible for qualitative interviews. In examining differences in the distribution of baseline pre-randomization characteristics between eligible participants who completed the qualitative interviews (*n* = 100) and participants who did not complete the qualitative interview (*n* = 65), we found no significant differences in the intervention condition, anxiety and depression symptoms, level of functioning, disability, gender, education level, number of chronic conditions, smoking status, self-rated physical health, and self-rated mental health. We also examined differences in COVID-19 diagnosis and hospitalization distribution between these two groups using self-reported information from the COVID-19 follow-up assessment. Compared to those who did not complete the qualitative interview, participants who responded to the qualitative interview were older, more likely to be Asian or Pacific Islander, less likely to be Hispanic or Latino, more likely to be foreign-born, and more likely to be married or cohabitating. We also found that participants who responded to the qualitative interviews were less likely to be diagnosed with and hospitalized due to COVID-19.

### 2.2. Procedure

Research assistants trained by Ph.D.-level researchers conducted semi-structured interviews with racially/ethnically diverse older adults. They conducted interviews by telephone, lasting roughly 60–90 min. The research team developed a set of open-ended questions focused on the following topics: (1) participants’ attitudes toward the newly released COVID-19 vaccines, (2) participants’ willingness to receive the vaccine, and (3) reasons why they or other people are not willing to receive vaccines. The questions used in the interview to prompt these topics were:

1. What is your attitude towards the vaccines released for COVID-19?
2. Are you willing to be vaccinated in the coming months?
3. Some people don’t want to get the vaccine. Why do you think that is?

The present study’s questions align with previous studies utilizing qualitative approaches to elucidate attitudes and hesitancy toward COVID-19 vaccination in various populations. Previous studies have asked questions regarding participants’ attitudes toward the COVID-19 vaccine [19,33,37], concerns they or other people might have about the COVID-19 vaccine [19,32,38], and if they would accept the COVID-19 vaccine [37].

The Mass General Brigham Institutional Review Board approved this study.

### 2.3. Analysis

Research staff transcribed the interviews verbatim, with several checked by a different staff member to validate the quality and accuracy of the transcription. Transcripts were coded using Dedoose [39], a software platform developed to analyze qualitative data. The research team used a thematic analysis approach consisting of 5 phases: familiarization with the data, generating codes, coding transcripts, generating, and reviewing themes, and describing these themes [40]. Utilizing the thematic analysis framework, the research team first independently read through the first few interviews for data familiarization. Researchers then met with two coders trained in qualitative interviewing and coding using standardized materials based on Braun and Clark’s work [40]. Coders read through three interviews and met with the research team to agree on initial themes used to develop preliminary codes; preliminary codes were used to develop the first codebook. Trained coders blind-coded the three interviews independently, meeting with the research team to discuss codes and resolve differences, with the researcher serving as a tiebreaker. Next, three additional interviews were blind-coded independently using the preliminary codebook; coders kept memos to log differences with fit and additional themes for consideration. Upon review with the research team, codes were discussed and resolved, additional codes were considered, and codebooks were modified and finalized to integrate and organize final codes. The resulting final codebook was uploaded into Dedoose for coding and analysis. After each transcription was double-coded, coders met to resolve the incongruencies and reach a consensus. When coding consensus reached 95%, the coders switched from double-coding independently to single-coding. The team identified a set of themes that emerged after the coding process. These themes were subsequently discussed and refined with the research team members.

## 3. Results

### 3.1. Sample Overview

A total of 100 participants were interviewed. The average age of participants was 77 years old (see Table 1 for detailed sociodemographic data of the interviewed participants). Most participants were female (*n* = 84), born outside the United States (*n* = 82), and living in Massachusetts (*n* = 71). Almost half of the participants were Chinese (*n* = 48). Out of all 100 interviews, most were conducted in Chinese (*n* = 48), either Mandarin (*n* = 28) or Cantonese (*n* = 20); followed by Spanish (*n* = 31) and English (*n* = 21).

Three main themes were identified through thematic analyses. The first theme reflects diverse older adults’ mixed attitudes toward the COVID-19 vaccine, centering mainly on matters of trust and information being consumed. The second theme highlights how health-related factors were most influential in vaccine uptake, specifically concerns about the uncertainty of potential side effects weighed against the fear of dying. The final theme focused on non-health-related, systemic factors influencing vaccine uptake, including the impact of trusted figures in influencing vaccination decision-making in older adults.

### 3.2. Theme 1: Most Older Adults Showed Mixed Attitudes toward the COVID-19 Vaccine, Strongly Associated with Trust or Mistrust toward the Host Country’s Healthcare System, as Well as Information or Misinformation They Deemed True

Many participants discussed and regarded the vaccine with optimism. Several mentioned that they trusted the vaccine and were willing to be vaccinated or had already been vaccinated. These participants described the vaccine as good, beneficial, and effective and advised others to become vaccinated. They expressed views that the vaccine would benefit, protect, and relax them, even suggesting that vaccination should be mandatory, as it would be essential to stop the pandemic:

P5 (Chinese): “I think it is good. People should trust it. Everyone should quickly get vaccinated. In that way, I would feel safer and don’t have to worry about going outside… This [COVID-19] has already happened. We should face it. What else can we do to fix it? The solution now is to get vaccinated and trust scientifically based remedies.”
P25 (White): “I’ve gotten my vaccine, and I feel it has given me some freedom. I trust that the vaccine is good.”
P50 (Latinx): “I am vaccinated, and I feel, inside, safer or more relaxed […].”

However, despite positive views regarding the vaccine, some older adults also revealed perceptions of the vaccine that reflected mistrust of the organizations developing and administering them. Sentiments of mistrust included viewing the vaccine as fraudulent and as a means of experimentally mistreating vaccine takers, using them as test subjects for non-pandemic matters. Participants often expressed concerns about what they viewed as quick and hasty development of the COVID-19 vaccine and a lack of sufficient testing. These statements contrasted with other participants’ declaring the COVID-19 vaccine as imperative in overcoming the pandemic.

P3 (Black): “There’s a lot of seniors I know they said they’re using them as guinea pigs. You know, that’s why I think they don’t want to get it.”
P29 (Latinx): “They don’t believe in the vaccine. They think it’s a game, that that’s a lie.”
P17 (Other): “A lot of people think it’s like a poison you’re putting in their arm.”
P70 (Chinese): “They may be just afraid that the vaccines are not reliable.”
P35 (White): “You know, we-were guinea pigs here. I think they’re poison.”

### 3.3. Theme 2: Health Concerns and Underlying Medical Conditions Were the Most Influential Factors in the Decision-Making Process of Older Adults about the COVID-19 Vaccine

Participants reported that concerns about health and underlying medical conditions drove them to get vaccinated. Some participants were encouraged toward vaccination after being informed about the possible outcomes of a COVID-19 infection. Others initially hesitated due to underlying medical conditions, such as cancer, asthma, and allergies, and feared a potential interaction between the vaccine and their ongoing comorbidities. Through thematic analysis, various subthemes centering on health concerns as factors that may impede or promote vaccination were identified.

#### 3.3.1. Theme 2.1: Some Older Adults Are Getting Vaccinated Because They Are Afraid to Contract the Virus and Experience Its Associated Symptoms

Some initially hesitant participants ultimately decided to get vaccinated, while others were eager to become vaccinated as soon as they were eligible. These older adults described vaccination as a means of protection, prevention, and solution to end the pandemic, expressing feeling safe after taking the vaccine. Many voiced fears of contracting COVID-19, which prompted their vaccine uptake, and others noted that vaccination could potentially reduce the severity of the virus’ symptoms if they were to contract it.

P1 (American Indian): “No, I’m gonna take it anyway because I don’t want to get sick, with people around the school and everything.”
P79 (Chinese): “I am willing to be vaccinated because the vaccine can increase my immunity against the virus. It should be good for my health, and it is protective. After being vaccinated, at least I won’t get sick, or even if I get the virus, the symptoms will be milder.”
P57 (Latinx): “Vaccination because if you get COVID, it doesn’t hit you as hard, and you can survive.”

#### 3.3.2. Theme 2.2: Some Older Adults Hesitate to Get Vaccinated Because They Fear the Possible Side Effects of the Vaccine and How Potential Side Effects Might Affect Their Current Medical Conditions

Even though many participants were willing to get the COVID-19 vaccine, some discussed reasons they and others would not want to be vaccinated. When asked about possible hesitations in receiving the vaccine, older adults’ responses mainly centered on fear of side effects or fear of death resulting from the vaccine. This fear was described by participants in the context of their pre-existing health concerns and possible interactions between the COVID-19 vaccine and their health. Morbidities such as cancer, heart disease, poor physical condition, and frequent allergic reactions were highlighted. Concerns such as infertility, dizziness, fever, death from some reaction, and even the possibility of contracting the virus through the vaccine also contributed to vaccine deterrence.

P7 (Chinese): “I am afraid that I might have bad reactions after I get vaccinated. I am worried that the vaccine might worsen my health.”
P19 (Latinx): “(People are afraid of getting the vaccine) Because people die, they see that they are dying after getting this vaccine.”
P22 (White): “Yes, because of my personal medical history. I always get very sick when I get a viral illness, or I’ve gotten other vaccines, so I was hesitant because of that.”
P7 (Chinese): “I saw it from the newspaper or other places that some people died because of the bad reactions they had after vaccination.”
P56 (Latinx): “I am almost decided not to get it, but if I have no choice, I’ll get it. Also, I’m allergic […] to various medications. I need to be thoroughly tested, and if I don’t have a choice, I’ll get it.”
P51 (Latinx): “I think it’s for fear that they have a bad reaction and die. The real fear is of dying.”

### 3.4. Theme 3: Older Adults Underlined Systemic Barriers and Trusted People’s Attitudes and Circles as Additional Factors Influencing Vaccine Uptake

Older adults also discussed how lack of accessibility, fear of government control, general information or misinformation spread through social media or word of mouth, and others’ mistrust of the vaccine deterred vaccine uptake. Some of these sentiments are gathered in excerpts in Table 2.

On the other hand, older adults highlighted how seeing others getting vaccinated ultimately convinced them to get the COVID-19 vaccine. Also contributing to vaccine uptake was the desire to return to social life or the ability to travel or gather. Table 3 highlights participants’ responses on factors contributing to COVID-19 vaccine uptake.

#### 3.4.1. Theme 3.1: Communication with Healthcare Providers, Religious Leaders, and Family Members Influence Older Adults’ Decision-Making on the Uptake of the COVID-19 Vaccine

The attitudes of trusted individuals played an important role, both encouraging and deterring vaccination among participants. Although most physicians encouraged vaccination, some discouraged their patients from getting vaccinated by showing apprehension or uncertainty about the vaccine’s efficacy. Similarly, family members’ attitudes toward the vaccine both facilitated and dissuaded vaccine uptake in older adults. Moreover, participants often remarked on the influence of religious leaders’ attitudes toward the vaccine on the uptake of the vaccine. Table 4 illustrates quotes that reference these factors.

#### 3.4.2. Theme 3.2: Chinese Participants Mentioned Getting the COVID-19 Vaccine to Habitually Comply with Government Policy

Chinese older adults often referenced following the government’s orders as a determining factor in getting vaccinated, stating that they were used to following government policy and mandates, a habit stemming from sociopolitical norms in China. As an additional insight, some Chinese participants voiced how they believed Americans are used to freedom and liberty, and thus daring to go against government policy and public health guidelines on vaccine efforts and mandates. Aside from Chinese participants, no other racial/ethnic minority group referenced cultural elements tied to their sociopolitical conventions.

P72 (Chinese): “I think Chinese people are used to listening to the Communist Party anyway. They are more obedient. For example, if they want you to be vaccinated, I’d say that you may die anyway, no matter whether you get vaccinated or not. […] I still believe in government and believe in science. […] It seems that Americans are used to freedom. They are like this. Chinese people are more obedient.”
P86 (Chinese): “The government asked us to vaccinate, so we did it to support their policies.”

Participants discussed the importance of public officials and the President being more actively involved in public health messages supporting vaccine safety and efficacy.

## 4. Discussion

The current study examined attitudes and decision-making processes regarding COVID-19 vaccine uptake from the perspectives of diverse primarily immigrant older adults in the United States. Results from the research team’s thematic analyses highlighted three main themes. First, there were mixed attitudes and opinions toward the COVID-19 vaccine, which were dependent on cultural and informational factors, and similar to the findings of other studies [26,41,42]. Second, individuals’ health concerns influenced their decision-making to get the vaccine, analogous to what has been established with other older populations [43]. Last, systemic factors (e.g., accessibility issues) impacted vaccine hesitancy. Nonetheless, mixed perceptions of the COVID-19 vaccine were evidenced in participant interviews, underlining various factors taken into consideration when deciding to get the COVID-19 vaccine. Immigration-related and cultural factors included trust or mistrust of the healthcare system of the host country, as well as general confidence in their elected officials. Trust in authorities, healthcare systems, and the government are central to vaccine uptake [15], an area that merits further research.

Although previous literature has shown that vaccine hesitancy among racial/ethnic minorities is greater than among Whites [44], most diverse older adults in this study showed positive attitudes toward the COVID-19 vaccine after weighing the risks. Some participants voiced hesitancy toward the COVID-19 vaccine due to their fears of side effects, including death, if the vaccine interacted with their underlying medical conditions. These findings are consistent with previous research that found that vaccine uptake is mainly due to interest in personal protection from COVID-19. At the same time, concerns about possible side effects are the main factors contributing to vaccine hesitancy [10]. In the United States, racial/ethnic minority individuals most frequently worry about the long-term effects and adverse reactions of getting vaccinated [44], with Black and Latinx individuals having greater concerns about severe side effects [45]. Future research should explore cultural and contextual factors that explain the subgroup variations driving these differences.

Participants also discussed mistrust as a reason for vaccine hesitancy. Previous research has shown that low levels of trust in the government contribute to a lower willingness to be vaccinated [46]. Historical injustices in healthcare against racial and ethnic minorities and structural inequities that continually disadvantage marginalized communities drive mistrust [47]. Mistrust of government systems among immigrant communities might also relate to fear of deportation [48]. Public health officials should, therefore, build trust, as Quinn and Andrasik [49] suggested, with marginalized communities, working with community-based organizations rather than solely or primarily communicating through government-based systems [50]. Other cultural or sociopolitical underpinnings of vaccine decision-making found in this study include Chinese participants attributing their habitual obedience to government policy and subsequent vaccine uptake to political norms in China. Future research exploring social, political, and cultural factors in health decision-making is crucial to better inform and develop effective dissemination and engagement efforts [51] that reach diverse populations and subsequently dismantle health disparities in racial/ethnic minorities.

Diverse older adults identified family members as key agents in their decision-making regarding vaccine uptake. This aligns with previous research showing that family plays an important role in health-related decision-making for racial and ethnic minorities [52,53]. Family is a cultural value in racial and ethnic minority communities [54,55]; this, along with findings that family influences health decision-making, highlights the importance of considering diverse communities’ cultural values, such as involving the family in public health outreach efforts to diverse communities. Aside from the government, older adults in our study often relied on specific people for their vaccine information, including healthcare providers and religious leaders. Per our findings, some church leaders urged their congregants not to get the vaccine. Religious leaders are central and trusted people in communities [56], particularly among immigrant and racial/ethnic minorities [57,58,59]. Therefore, public health officials should develop projects that involve well-known religious leaders, as well as faith-based health interventions [60] to combat hesitancy that may deter COVID-19 vaccination.

An important systemic factor influencing vaccine deterrence cited by older adults in our study was lack of access. Challenges of access fundamentally account for under-vaccination [61], with factors such as infrastructure and health systems affecting the vaccine’s fair distribution [46]. Some participants were bedridden and depended on vaccination efforts coming to their homes, while others had to travel long distances to get the vaccine. Structural inequities, such as lack of transportation and convenient locations to vaccinate against COVID-19, obstruct vaccination in racial and ethnic minority communities [62]. Upon initial development and distribution of the COVID-19 vaccine, recommendations for ensuring high vaccine uptake in immigrant communities included setting up vaccine clinics in convenient community settings, such as schools or workplaces [48,63].

Although most diverse older adults reported willingness to be vaccinated for COVID-19, damaging systemic barriers, health-related fears, and attitudes in their social networks drive their decision-making processes. Attributing vaccine hesitancy solely to people’s perceptions and informational inadequacy downplays the role of systemic flaws, such as lack of accessibility and mistrust of healthcare and government systems. By placing the responsibility of becoming ‘less hesitant’ on the minoritized individual [64], the acknowledgment, problematization, and subsequent addressing of systemic flaws are wrongly minimized. Vaccine hesitancy must be contextualized as failures of healthcare and government systems in addressing systemic and logistical barriers that hinder vaccination efforts and uptake. Future research should explore strategies and policies that address systemic barriers and mistrust. Additionally, to avoid putting the onus on marginalized individuals to overcome injustices historically and structurally placed upon them, public health officials should tailor their vaccination efforts to the needs of diverse populations. This could help ensure that these programs consider and address previous sociohistorical experiences and current systemic barriers impacting racial and ethnic minorities.

The results must be reviewed in light of some limitations. While a strength of our study is that we could interview 100 diverse, mostly immigrant participants recruited from four states and a territory with different contexts, backgrounds, and languages, our sample of participants was limited in certain respects. Those who did not respond to the qualitative interview were more likely to be Hispanic or Latino, U.S.-born, more likely to be non-married, and less likely to be diagnosed with and hospitalized due to COVID-19. This has implications in that qualitative responses could more likely be of participants that were healthy and less seriously impacted by the COVID pandemic. Although we sought to encompass perspectives of different immigrant and minority communities in the United States, we also consider our data limited by including only a few Black participants and a sole American Indian participant, both part of widely marginalized groups in the U.S. We also confronted limitations when examining Chinese testimonials, since analyses were based on English translated versions of these insights by bilingual Chinese research staff, potentially losing nuances. Nonetheless, data obtained from these interviews, as well as through our thematic analyses, can be helpful in future planning and implementation of vaccine hesitancy interventions and policies tackling systemic barriers impacting health decision-making among older immigrant populations.

## 5. Conclusions

In communities of racially/ethnically diverse older adults, accessibility to accurate and consistent information is needed to combat vaccine mistrust and perceptions of the COVID-19 vaccine as unreliable and ineffective. We believe efforts tackling concerns of potential side effects from the COVID-19 vaccine should be prioritized. We also found that vaccination outreach efforts to promote COVID-19 vaccine uptake should publicly involve family members, physicians, and religious leaders as ambassadors of the vaccines. Moreover, these efforts should tackle systemic shortcomings, such as lack of accessibility and mistrust, observed in diverse communities. Public policy that addresses transportation issues and work schedules should be further considered.

## Figures and Tables

**Table 1:** Sociodemographic information of interviewed participants (*N* = 100).

Descriptor	*n* (%)
Age	
60–64	4 (4.00%)
65–74	35 (35.00%)
75+	61 (61.00%)
Gender	
Female	84 (84.00%)
Male	16 (16.00%)
Place of birth	
Outside the United States	82 (82.00%)
United States	18 (18.00%)
Race or ethnicity	
Chinese	48 (48.00%)
Latinx	30 (30.00%)
White	11 (11.00%)
Black	5 (5.00%)
Other	5 (5.00%)
American Indian	1 (1.00%)
United States state or territory	
Massachusetts	71 (71.00%)
New York	16 (16.00%)
Florida	7 (7.00%)
Puerto Rico	6 (6.00%)
Language of conducted interview	
Spanish	31 (31.00%)
Mandarin	28 (28.00%)
English	21 (21.00%)
Cantonese	20 (20.00%)

**Table 2:** Representative quotes of factors deterring vaccine uptake.

Factors Deterring Vaccine Uptake	Illustrative Quote
Accessibility	“From the city, they were supposed to come to give me the vaccine. They didn’t come. I called this week, and I was told they still don’t know. The other agency where my kids put me on the list called and said to be on the lookout because they would call to come to give me the vaccine. They haven’t come, either. […] Those of us who are bedridden need someone to come give us our vaccine in bed, and they’ve forgotten about us.” (P61, Latinx).
Fear of government control	“There are many people who say that this is a chip that they put in you.” (P27, Other).
Misinformation	“Because of people’s comments, the news that it was going to cause some effects to many people. I even heard that for couples that got the vaccine, the woman would not be able to have children. They would say many things that scared you.” (P31, Latinx).


**Table 3:** Representative quotes of factors facilitating vaccine uptake.

Factors Facilitating Vaccine Uptake	Illustrative Quote
Others getting vaccinated	“I saw the President got vaccinated too, so I got it myself. And whoever got the vaccination had no issues, so I got vaccinated myself.” (P46, Chinese).
Return to social life	“[…], that’s why I got the shot. I hope that within the next couple of months they’ll open back more, like the library, and stuff like that, so we can get out more.” (P43, White).
Travel	“I want to travel-I want to I want to go home, to my other home, and the only way I can do that safely is to get vaccinated.” (P4, Black).

**Table 4:** Representative quotes of trusted figures’ influence on vaccine uptake.

Attitudes Influencing Vaccine Uptake	Illustrative Quote
Familial intervention	“I felt that [my daughters], that they wanted me to get vaccinated […]. I don’t have any reason to make my daughters suffer. […] and I told him, well I accept it, and yes, I did get vaccinated.” (P53, Latinx).
	“My eldest son doesn’t want me to get vaccinated. He tells me no, he tells me mom, not that.” (P24, Latinx).
	“And plus, my kids were concerned about me not having the shot they were encouraging me to take it.” (P40, White)
Physician’s guidance	“So, I was afraid to get the vaccine because of my cancer. But then I sought information with [name], Dr. [name]. I called her, and she guided me. She told me that I was fine to get it. I talked to my cancer doctor. He said I was fine. I should get it. So, I said OK. Now I’m going to get it [the COVID-19 vaccine].” (P31, Latinx).
	“What do I think about the vaccine? I don’t trust them at all, and neither do my doctors. […] My doctor didn’t want me to [get vaccinated] because she wanted me to wait for a certain vaccination that was supposed to be coming here, but it’s taking too long. So now, she said I have to get vaccinated by the ones here that she feels are very bad for me, […]. If you read the science of it that is never put out there, but I’ve read it from doctor’s note—it’s so dangerous for people, their neurological and coronary conditions.” (P35, White).
	“Well, the doctor gave it [the vaccine] confidently, more or less confidently.” (P58, Latinx).
	“For example, my wife was against the vaccine, but she’s now following the rules that the doctor told her.” (P54, Latinx)
Religion and religious leaders	“I saw in the news a cult, a church that the minister or I don’t know what he was, he told the followers not to get the vaccine.” (P59, Latinx).
	“Churches have a lot to do with it. Yes. Because a lady says, ‘my church says no, that [the vaccine] is not necessary’.” (P16, Latinx).
	“I got the vaccine with faith in the Lord, in God, that nothing is going to happen to me, and I will get all the ones that they give me. […] I’m not scared. I got it very calmly, with faith.” (P62, Latinx)

## Data Availability

The data presented in this study are available upon reasonable request. The data are not publicly available due to the sensitivity of the data and the author’s agreements with the Institutional Review Boards of the participating institutions. Reasonable data access requests should be directed to Sheri Markle at smarkle@mgh.harvard.edu.
